# Spontaneous chiral symmetry breaking in early molecular networks

**DOI:** 10.1186/1745-6150-5-38

**Published:** 2010-05-27

**Authors:** Ran Kafri, Omer Markovitch, Doron Lancet

**Affiliations:** 1 Department of Molecular Genetics, Weizmann Institute of Science, Rehovot 76100, Israel; 2 Department of Systems Biology, Harvard Medical School, 200 Longwood Avenue, Boston, MA 02115, USA

## Abstract

**Background:**

An important facet of early biological evolution is the selection of chiral enantiomers for molecules such as amino acids and sugars. The origin of this symmetry breaking is a long-standing question in molecular evolution. Previous models addressing this question include particular kinetic properties such as autocatalysis or negative cross catalysis.

**Results:**

We propose here a more general kinetic formalism for early enantioselection, based on our previously described Graded Autocatalysis Replication Domain (GARD) model for prebiotic evolution in molecular assemblies. This model is adapted here to the case of chiral molecules by applying symmetry constraints to mutual molecular recognition within the assembly. The ensuing dynamics shows spontaneous chiral symmetry breaking, with transitions towards stationary compositional states (composomes) enriched with one of the two enantiomers for some of the constituent molecule types. Furthermore, one or the other of the two antipodal compositional states of the assembly also shows time-dependent selection.

**Conclusion:**

It follows that chiral selection may be an emergent consequence of early catalytic molecular networks rather than a prerequisite for the initiation of primeval life processes. Elaborations of this model could help explain the prevalent chiral homogeneity in present-day living cells.

**Reviewers:**

This article was reviewed by Boris Rubinstein (nominated by Arcady Mushegian), Arcady Mushegian, Meir Lahav (nominated by Yitzhak Pilpel) and Sergei Maslov.

## Background

The derivation of chemical reactions that spontaneously generate an excess of one enantiomeric form (i.e. one of two stereo-isomers of an asymmetric molecule endowed with the property of handedness or chirality, and mutually related by mirror symmetry) has been a central ambition of numerous theoretical and experimental studies [1-6]. The challenge is to depart from a racemic mixtures (having equal amounts of both isomers), and reach enantiomeric excess without the aid of external chiral selectors. Thus (reviewed in [5]), some authors have proposed that a catastrophic symmetry breaking event was necessary to explain why in a class of biomolecules (e.g. amino acids) all members have the same chiral configuration. Energy imbalance of enantiomers due to a lack of antimatter parity, or enantioselective breakdown by circularly polarized light from space was invoked. It was argued, however, that a viable statistical model could replace these cosmic explanations, a model merely based on evolutionary properties such as propagation and competition.

Indeed, several studies invoked relatively simple kinetic models, in which initial racemates with fluctuations undergo reactions that lead to chiral purity, thus demonstrating the plausibility of symmetry breaking in a non-equilibrium regimen [5,7-9]. Many such treatises assume that chiral selection has occurred under abiotic conditions, and preceded (or even served as a prerequisite for) life's origin. Among these are models that involve bifurcation in small molecules [8-10] In parallel, systems were reported that involve polymerization [11,12] as well as interactions within crystals (reviewed in [12]). The basic principles that guide such papers include the notion of statistical fluctuations, namely that in an ensemble of asymmetric molecules of a given type, there will always be an excess of one enantiomer, particularly apparent in small ensembles, and that such fortuitous excess may be greatly amplified by catalytic or replicative reactions [8]. The present paper rests on such view, and attempt to provide a novel concrete and quantitative framework for its realization.

Life is believed to have emerged by self organization processes occurring within a random and highly heterogeneous chemical environment [10,13]. One of the hallmarks of some other prebiotic evolution studies is the assumption that homochirality (the prevalence of only one of the two chiral isomers), widespread in present-day life, has emerged as part of the processes that led to cellular life [14-19]. For example, it has been argued [14] that information theory conclusions can explain why chiral building blocks, as well as sets thereof, are necessary in living systems, and that simplest forms of life likely constituted autocatalytic reactions such as the Soai reaction [4] where a chiral product acts as a chiral catalyst for its own production.

It is thus crucial to ask how chiral symmetry breaking could become possible under the conditions that prevailed at the early emergence life (see for example [19], [20] and references thereof). By one school of thought, the origin of life is proposed to have occurred through kinetically self organizing processes controlled by defined chemical interaction networks [21-26]. In this respect, models accounting for life's origin could be helpful for the understanding the generation of chiral purity. A case in point is the Graded Autocatalysis Replication Domain (GARD) model we have developed [23,27-34]. The model entails a chemically diverse set of mutually catalytic amphiphiles that spontaneously aggregate to form molecular assemblies (e.g. micelles, a "Lipid World" scenario [29], see also [35]). It was shown that such assemblies often self-organize into kinetically stable mutually catalytic networks, termed composomes, display homeostatic growth and reproduction-like processes, as well as a limited capacity for selection [30,36-38]. One unique aspect of GARD, with respect to other proposed models, is that the life-like chemical networks are not *a-priori *engineered for this specific purpose, but rather spontaneously emerge.

GARD nominally belongs to a set of models that assume that early prebiotic evolution took place completely in aqueous solution, in distinction from scenarios that point to a liquid-solid interface as indispensable for life's origin. Mineral interfaces are presumed to have provided catalysis, compartmentalization and sometimes also a free energy source ([39-41] and refs thereof). However, all three such aspects are provided by in GARD and similar models via lipid-water interfaces. Furthermore, lipid-based models are not contradictory to the involvement of solid interfaces, which lipid assemblies could interact.

Since GARD composomes were demonstrated to select idiosyncratic compositions from a molecularly diverse external environment [23,42,43], it is an intriguing possibility that chirally biased assemblies and composomes may spontaneously emerge from a racemic medium. Such notion is in line with experimental demonstrations showing spontaneous separation of racemates in crystals monolayers and amphiphilic aggregates [7,8,44-50].

We ask here whether a collection of simple organic chiral amphiphilic molecules could assemble into a reaction network that, in the absence of any external chiral perturbation, would catalyze preferential enantiomer selection. In contrast to previous theoretical models, we do not engineer a reaction network that is designed to generate symmetry breaking, but rather apply random chemistry by generating a large diversity of molecules and chemical reactions. Our results suggest that random catalytic networks may self organize into chirally selected compositions, which in addition portray homeostatic growth and dynamic properties akin to self reproduction.

## Results

### Chiral compositional dynamics shows enantiomeric selection

**Figure 1:**
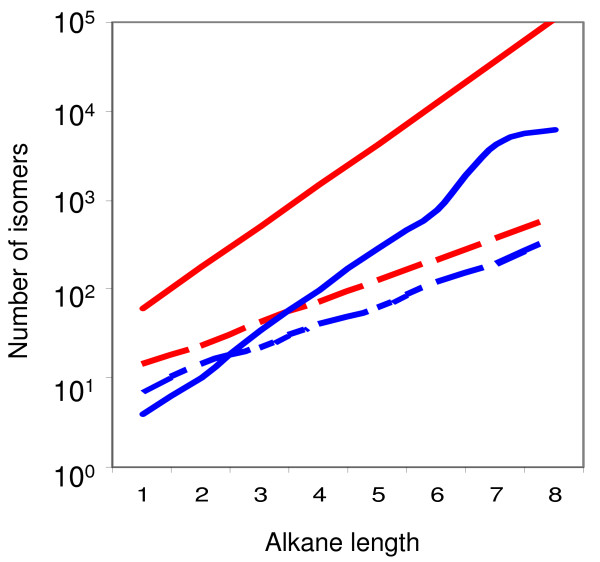
**The number of possible chiral (solid) and non-chiral (dashed) isomers as a function of the number of carbons in an Alkane**. Red represents a case where one carbon is replaced by a hetero atom, and blue denotes a case of no hetero atom. Data is taken from [54]. This figure demonstrates that for sufficiently complex molecular structures it is a good approximation to assume that all molecules are chiral [51-55].

**Figure 2:**
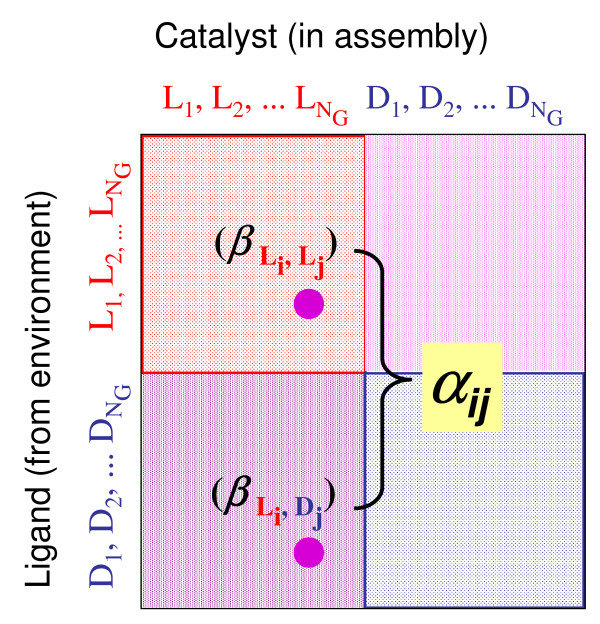
**An illustration of a 2*N*_*G*_ × 2*N*_*G *_*β *matrix and the value of *α***. Note that the two blocks along each diagonal have identical values of the affinities (β^*LL *^= β^*DD *^and β^*LD *^= β^*DL *^).

To investigate the network behavior of collection of chiral amphiphilic molecules, we employ the GARD model (Methods) in ways that accommodate chirality (Chiral-GARD or C-GARD). We thus consider an environment with a population of *N*_*G *_types of asymmetric molecules in a racemic mixture that contains equal amounts of the D and L optical isomers of each molecule type. For sufficiently complex molecular structures it is justified to assume that essentially all molecules are chiral [5,51-55] (Figure 1). All 2 × *N*_*G *_molecule types are treated as different compounds with different kinetic parameters, keeping in mind that they actually constitute 100 enantiomer pairs (Methods and Figure 2).

**Figure 3:**
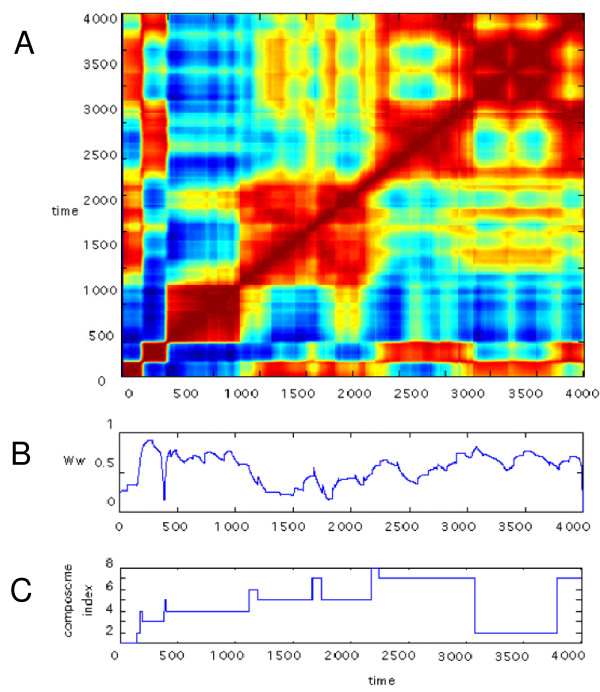
**A, Correlation diagram between pairs of time points during a C-GARD simulation, using Eq. 1**. *H *= 1 and *H *= 0 (Eq. 2) are respectively marked by red and blue, and 0 <*H*< 1 is shown by intermediate rainbow colors. Parameter values used are: *k*_*f *_= 5 × 10^-2^, *k*_*b *_= 5 × 10^-3^, *N*_*G *_= 100, N_max _= 200, *ρ *= 10^-2 ^and *σ*_*ε *_= 6; **B**, The time-dependent behavior of weak enantiomeric selection (*W*_*w*_, Eq. 7) during this simulation; **C**, Composome assignments for the assemblies analyzed in B (see Methods). Note that each composome tends to have a distinct *W*_*w *_value.

We asked whether in C-GARD, composomes may display enantioselection, in analogy to the chemical selection seen in the dynamics of the GARD model [28-30,32,34]. For this, we employed a measure termed here "weak enantioselection", denoted *W*_*W *_(Methods, Eq. 7). The top panel of Figure 3 shows a correlation diagram for all 4000 time points in one such simulation. Importantly, appreciable non zero values of *W*_*W *_occur at most time points, suggesting that many of the compositions are symmetry-broken. We note that at high *W*_*W *_values, each of the compounds has a high enantiomeric excess, although not necessarily in the same handedness for all compounds ("Strong enantioselection" *W*_*S*_≈0, Eq. 8). The simulation further displays the appearance of distinct composomes along the time axis, and it is apparent that different composomes have different average *W*_*W *_values.

**Figure 4:**
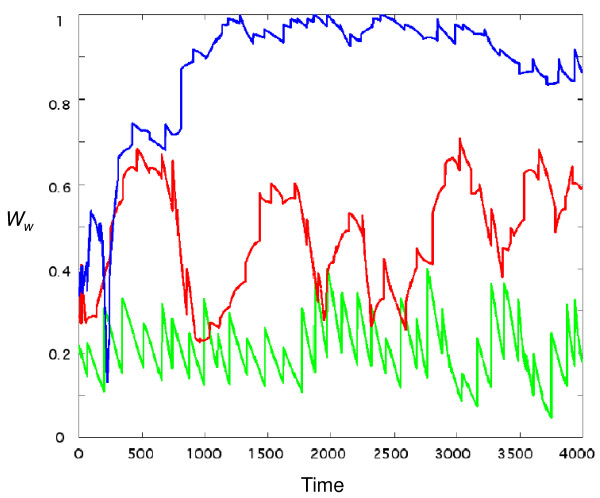
**Time dependence of *W*_*W *_for three simulations (*σ*_*ε *_= 1 green, *σ*_*ε *_= 3 red, *σ*_*ε *_= 6 blue)**. Other parameter values are as in Figure 3. It is seen that the typical *W*_*W *_increases with *σ*_*ε*_, because higher enantiomeric discrimination (*α*) values are allowed. The saw-tooth patter arises from growth-fission cycles of the C-GARD assembly.

**Figure 5:**
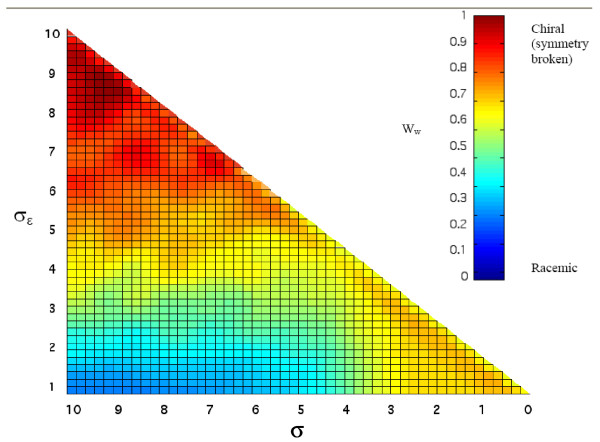
**Two dimensional analysis of the dependence of average *W*_*W *_on λ and *σ*_*ε*_**. Data are based on 60 simulations, each with 4000 time steps, with the same parameters as in Figure 3, except N_max _= 300. Figure produced from ref[86].

Figure 4 shows a typical variation of *W*_*W *_as a function of time for several values of *σ*_*ε *_, a parameter determining the distribution of the enantiodiscrimination *α *(Eqs. 4 and 6). Larger values of *σ*_*ε *_result in increasing *W*_*W *_to the point of reaching nearly complete weak enantioselection (*W*_*W *_→1), as also seen at the top apex of Figure 5. The observed *W*_*W *_fluctuations at low *σ*_*ε *_result from the assembly size variation with assembly growth and split. Such fluctuations are significantly reduced at higher *σ*_*ε*_, indicating compositional stability through the action of the catalytic network [21-23,29,30,56]. The variation on longer time scale seen for *σ*_*ε *_= 3 represent transitions among different composomes with varying degrees of chiral inhomogeneity.

Interestingly, significant non-zero values of *W*_*W *_are obtained even in the absence of any mutually catalytic effect, i.e. when both enantiodiscrimination and catalytic-potency-related parameter are low (*σ*_*ε *_and σ, respectively. See Eqs. 6 and 3) (Figure 5). This enantiomer excess is distinct from the presently described dynamic enantioselection, and relates to previously published predictions regarding statistical fluctuations at low molecular copy numbers [5,57-59]. Intriguingly, allowing high values of the rate enhancements suppress this statistical effect due to compositional bias whereby only a few molecules are present at low copy number (lower left apex of Figure 5).

**Figure 6:**
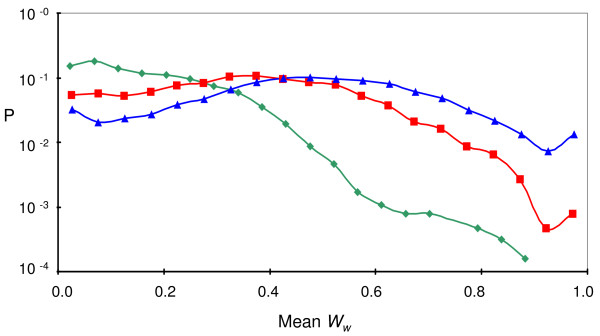
**Probability distribution of *W*_*W *_at different values of *σ*_*ε *_(colors as in Figure 4) based on 6000 simulations for each *σ*_*ε *_value**. Other parameter values are as in Figure 3, except *k*_*f *_= 10^-2^, *k*_*b *_= 10^-3^

Figure 6 shows a global analysis of the dependence of enantioselection on molecular enantiodiscrimination, integrating the results of 6000 different simulations. A probability distribution for the average *W*_*W *_values of assemblies is plotted for three different *σ*_*ε *_values. Increasing *σ*_*ε *_enhances the probability of assemblies to have high *W*_*W*_, yet even for the highest *σ*_*ε *_studied here (*σ*_*ε *_= 6) there is an almost even chance for assemblies to show high or low *W*_*W*_. This may indicates a stochastic effect: high enantiodiscrimination is necessary, but not sufficient to lead to symmetry breaking.

### Antipodal composomes

**Figure 7:**
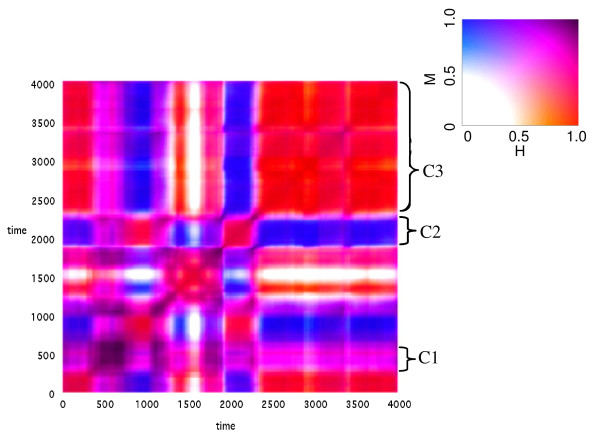
**A correlation diagram displaying the chiral compositional dynamics of C-GARD molecular assemblies with respect to both the similarity *H *(Eq.2) and the antipodicity *M *(Eq. 5)**. Three composomes (C1, C2 and C3) emerge in this simulation, with C2 and C3 being antipodes of each other. The 2-dimensional color scale is shown in the inset. Purple color indicates that at least one of the compositions is racemic, while off-diagonal blue shows appreciable antipodicity. Simulation parameters as the same as in Figure 6, except N_max _= 300 and *σ*_*ε *_= 0.8.

**Figure 8:**
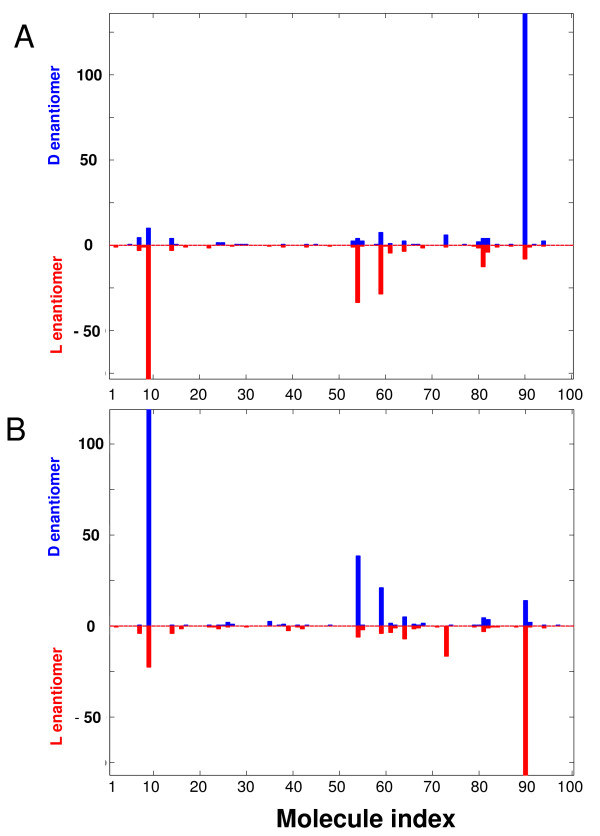
**Compositional bar-chart for the composomes C2 and C3 (Figure 7)**. The molecule index is serial number representation of the different compounds (1...*N*_*G*_), and D and L enantiomers are indicated by color and vertical direction. The Y axis is the count of a given molecule type within the composomal assembly.

We analyzed the symmetry properties of composomes that emerged in assemblies of chiral compounds. Figure 7 shows a correlation diagram in which different types of composomes are apparent, with symmetry properties highlighted by specific colors (inset). It may be seen that at certain time intervals highly asymmetric composomes appear while at different times the composomes are not symmetry-broken (C1). The figure further demonstrates the existence of antipodal composomes (C2 and C3, see compositional bar charts in Figure 8), each with broken symmetry, and showing mutual compositional mirror relationships as indicated by the off-diagonal blue areas (See Methods, Eq. 5). Interestingly, in certain cases network dynamics leads to abrupt transitions among composomes with different symmetry properties, including between antipodal composomes (e.g. near time point 2400, where a transition occurs directly between composomes C2 and C3).

### The dynamics of composome populations

We examined the capacity of the C-GARD model to portray evolution-like processes with selection of particular enantioselected molecular assemblies. To this end, we explored the competitive coexistence of numerous assemblies with different degrees of chiral symmetry. We simulate the time course of a single specific assembly and reconstructing from it an approximation for a time dependent population behavior, similar to a previously reported procedure [30]. The approximated population size of a given composome C_m _was computed as , where *t*_*m *_is a cumulative elapsed time period of assembly homeostatic growth/fission while being in the composomal state C_m _and *τ*_*m *_is the average time between fission events characterizing that composome. The rational for such a computation is that if a *bona fide *population of C-GARD assemblies were observed for a time period , the assemblies in composomal state C_m _will have undergone ~ fission events.

**Figure 9:**
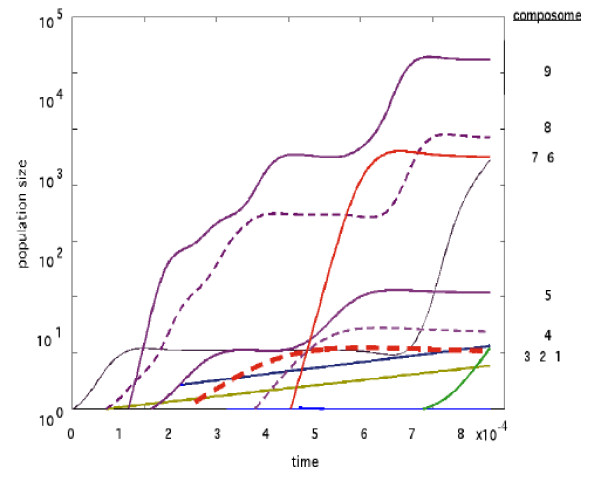
**C-GARD population dynamics emulating the competitive coexistence of composomes**. The time dependent population sizes for the different composomes were reconstructed in an approximated fashion from single assembly simulations as described in the text. Simulation parameters are as in Figure 3.

**Figure 10:**
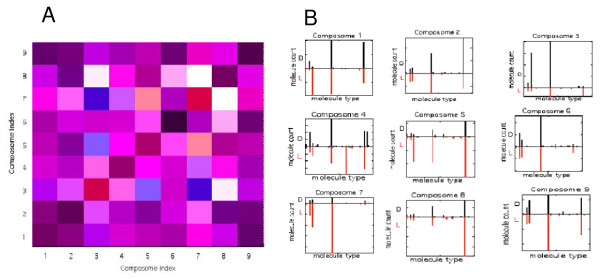
**A, the chiral relations among the composomes of Figure 9, with color scheme as in Figure 7; **B**, the compositional bar charts for all 9 composomes**.

Figures 9 and 10A shows results for a particular simulation. A competition-like behavior between the composomes is observed in which the relative proportions composomal states in the emulated population changes over the time course. This reflects the differences in composome "fecundity", represented by their time to fission τ_m_, manifested by the exponential nature of the population growth. We note that these simulations do not include events of assembly "death", reflecting an assumption that the simulated population size is sufficiently small to justify the buffered environment assumption [27,30].

Among all the composomes in Figure 9, only composomes 3 and 7 are appreciably enantioselected and the rest are racemic (Figure 10B). Other simulations show a majority of symmetry broken composomes (not shown). Interestingly, while composome 7 (red solid line) and composome 3 (red dashed line) are nearly antipodal, composome 7 which fortuitously first emerges later than composome 3, ends up with a simulated population size more than 100 fold larger towards the end of the examined time period. This is rationalized by the notion that each composomal state constitutes an ensemble of disparate, though similar compositions and small chance fluctuations may lead to noticeable differences in long-term dynamic behavior (cf. [60]).

## Discussion

### Multi-component kinetic enantioselection

The breaking of chiral symmetry in multi-molecular assemblies presented here constitutes a distinct class of plausible stereo-selective processes. An advantage over other models is by considering many pairs of enantiomers, thus offering systems-related enantioselective mechanisms. The same concept is manifested in a reported kinetic simulation of a simple network of replicating peptides [61], as well as in mutual interaction within molecular assemblies such as monolayers and 3-dimensional crystals ([12] and references thereof). It should be also noted that heterogeneous multi-component chemistry is more appropriate for describing early symmetry breaking processes, since prebiotic environments have likely been highly chemically diverse [27,33,62]. The C-GARD model presented here specifically assumes that symmetry breaking has occurred within assemblies of amphiphilic, lipid-like molecules. This, and similar concepts involving lipid molecular assemblies [63], stochastic aggregates [64] and crystalline arrays[12,16,45,50] have been previously explored by others.

Enantioselection is often portrayed as a non equilibrium kinetic process [3,9,61,65]. Many relevant kinetic formalisms [3,66-68] are based on the original model of Frank for spontaneous mirror symmetry breaking [9], which assumes a "chemical substance which is a catalyst for its own production and an anti-catalyst for the production of its optical antimer". Another set of models derives from the mechanism proposed by Kondepudi [8] with two autocatalytic achiral precursors whose dynamics result with homochirality. The C-GARD model presented here is also based on defining a set of kinetic equations for the different reaction paths. However, these kinetic equations are not designed *a-priori *to produce symmetry breaking. Rather, enantioselection spontaneously emerges in some of the molecular assemblies and is propagated by compositional homeostasis. The detailed mechanisms responsible for enantioselection may vary from one assembly to another and could conceivably include autocatalysis and mutual pairwise catalysis. Another advantage of our model is its ability to provide an estimate of the propensity of assemblies with different levels of symmetry breaking based on the kinetic equations derived from a statistical formalism based on molecular interaction [32,69-71].

We assume in the present analysis that all molecules in the C-GARD simulation are asymmetric (Figure 1). An interesting question is what would be the dynamic fate of symmetric compounds intermixed with a majority of asymmetric ones. Consider Eq. 4 for the case shown in Figure 2, for *i *= 17 and *j *= 11. Making molecule 11 symmetric, by the loss of a chiral center, which is assumed to have a minimal chemical effect otherwise, necessitates , where *X *denotes the symmetrized molecule. It may be rather safely assumed that (on average of many such cases) the value of  will be somewhere between  and , perhaps their geometric mean. Thus, symmetric molecules will not have an appreciable kinetic advantage or disadvantage.

### Symmetry breaking due to statistical constraints

An interesting aspect of the C-GARD model is a capacity to show a distinction between kinetically-controlled chiral selection, and apparent enantioselection in the absence of stereospecific molecular recognition. The latter arises due to statistical fluctuations relating to assembly size and chemical heterogeneity, as has been explored previously for polymer systems [5,57-59] and for generally diverse chemical systems [5]. In contrast, the chiral constitution of assemblies of kinetically-interacting molecules displays fluctuations between high and low values of *W*_*W*_, in agreement with the general characteristics predicted for non equilibrium symmetry breaking systems [61,65,72,73]. A mechanism for the symmetry breaking in non equilibrium systems was previously proposed [9] and additionally revised [9,65,73]. There is a relationship between this mechanism and the one depicted by C-GARD. Basically Frank's model constitutes a special case of a two dimensional C-GARD, in which the autocatalytic values in the *β *matrix are greater than 0 and the cross catalytic values are smaller than 0. An advantage of GARD is its generality, having less restrictive assumptions. Still, an appreciable symmetry breaking ensues, both for single and for multiple assembly simulations.

### Chirality and the origin of life

The C-GARD model predicts a considerable degree of chiral selection, but this happens at relatively high values of the enantiodiscrimination parameter *α*, as compared to the typical values obtained by a statistical analysis [74] of values reported in CHIRBASE [75]. This may be taken as an indication that assembly-based enantioselection could not have acted at the earliest stages of life's origin. CHIRBASE is, however, restricted mainly to small, relatively simple molecules. A study quantifying the capacity of random molecular structures to manifest enantioselection has predicted, through the use of random diffusion limited aggregates, a linear relationship between the size of the asymmetric structure and its enantiomeric discrimination [55]. This is also in line with analyses using string complementarily models, that generally show growing recognition capacity with increasing molecular sizes [69,70,76,77]. As more information is accumulated on the nature size and complexity of prebiotically-available molecules, better fine-tuning of the prediction of C-GARD will become possible. Furthermore, based on such relations between molecular size and enantiomeric discrimination it may become possible to predict the minimal size of prebiotic molecules that would generate sufficient symmetry breaking as inferred from the C-GARD model. Such analysis cannot however be presently performed based on CHIRBASE information, as this database does not have explicit display of molecular sizes.

A considerable number of publications perceive chirality not merely as a central characteristic of life but as a prerequisite for its emergence ([78] and references therein). This stems from the widely accepted notion that self-replication of biopolymers is essential for life's inception. Indeed, previous experiments [78,79] as well as in theoretical models [73,80] has indicated that a very high degree of chiral purity is required for successful polymer-based information transfer. Consequently, many works assumed that at some point in early earth history physical and/or chemical processes have led to pronounced symmetry breaking, which occurred in an inanimate environment and allowed the initiation of life processes. The alternative scenario presented here involves rudimentarily replicating compositional entities, such as GARD assemblies, independent of biopolymer templating. These afford a potential origin of chiral selection as part of the mutually-catalyzed accretion dynamics of non-covalent molecular assemblies. Once a sufficient degree of chiral selection is achieved, more elaborate informational biopolymers may become possible. Thus, the C-GARD model highlights the possibility that chiral selection is a result of, rather than a prerequisite for early life-like processes.

## Methods

### The GARD formalisms

The C-GARD model is built upon the GARD kinetic model [30,73]. A GARD molecular assembly, typically assumed to consist of amphiphilic molecules, grows by accretion within an buffered environment containing N_G _different molecule types, and undergoes a stochastic fission process designed to produce two (potentially similar) daughter assemblies. The assembly is represented by a compositional vector ***n***, such that the component *n*_*i *_depicts the number of molecules of type *i *within the assembly.

Assembly growth rate is governed by the following set of kinetic equations(1)

where *k*_*f *_and *k*_*b *_are the forward and backward reaction rates, *ρ *is buffered extraneous concentration of all molecule types, *N*_*G *_is the number of different molecule types, the assembly size is , and when the assembly size reaches the value N_max _we impose a stochastic split generating two progenies of equal size [30].

Equation 1 has an obvious steady state fulfilling  at , where * indicates a specific steady state value. We note that this is a stable uniform equilibrium steady state (with all n_i _equal), which is different from the dynamic quasi-stationary states [21,43] constituting the composomes. The dynamics involving periodic fission events averts the attainment of equilibrium, and induces continuous transitions among quasi-stationary states typical of GARD dynamics. Such behavior is in fact the result of stochastic small perturbations of the concentrations and rates, corresponding to GARD's life-like characteristics.

For evaluation of compositional similarity among different assemblies (e.g. at two different time points) we use the normalized dot product of the corresponding composition vectors (i.e. cosine of an angle between the two composition vectors) [29,30,32]:(2)

A similarity threshold of *H *≥ 0.95 is used in an iterative procedure to classify an assembly within one of several predefined composomes or, if necessary, to define a new composome. This procedure is akin to that which previously referred to as clustering of multiple instances of composomes into compotypes [81]. Mutual rate enhancement exerted by molecule type *j *on molecule type *i *is represented by the non-negative element *β*_ij _in an *N*_*G *_× *N*_*G *_matrix (Eq. 1). The choice of rate enhancement distribution characteristics in GARD is guided by an embodiment of the Receptor Affinity Distribution (RAD) formalism for catalytic activities [32,69-71], which is supported experimentally by analyses of immunoglobulin and phage display libraries [69]. The extension of RAD from affinities to catalytic rate parameters [32] derives from the relation between binding and catalysis governed by transition state theory and implies a lognormal distribution for the catalytic intensities *β*_ij_:(3)

Where μ and σ are respectively the mean and standard deviation of the distribution, and *γ *is a constant related to the subsite binding energy in the RAD model [69].

In the present embodiment we use a Poisson approximation with a single statistical parameter, λ, interpretable as the average number of successful intermolecular subsite recognition events in the RAD model (μ = λ and σ = λ^1/2^, [32] appendix). Except where otherwise indicated, we use λ = 6 which has been proven appropriate in a study that addresses GARD heritability properties [32].

### C-GARD and its symmetry properties

In a C-GARD simulation, half of the entries in the compositional vector represent the D isomers and the other half - the antipodal L isomers (Figure 2), thus leading to the definition of the compositional vector  where  and  are the counts of the two enantiomers of the molecule type *i *within the assembly. In C-GARD the assembly pre-fission size is .

We employ a parity principle of space inversion equivalence by requiring that the catalytic interaction coefficient for a given pair of molecules would be equal to that corresponding to their respective enantiomers, resulting in a chiral 2*N*_*G *_× 2*N*_*G *_*β *matrix (Figure 2). Parity-violating energy difference between enantiomers is excluded from the analysis as it is generally regarded too minute to account for macroscopic behavior [82-85].

A key property intrinsically associated with chirality is the ability of a chiral compound (e.g. L_i_) to differentiate between two encountered enantiomers, L_j _and D_j_, (Figure 2). Such capacity is represented by the value of the enantiodiscrimination factor *α*.(4)

To assess the degree to which two compositional vectors **n**_1 _and **n**_2 _are antipodal to each other, we define the antipodicity *M *as the similarity *H *(Eq. 2) between a composition **n**_1 _and,  the antipodal composition of **n**_2 _:(5)

where  and *vice-versa*. Note that since *M *is defined through the measure *H*, it is also normalized and obtains values ranging from zero to one, where the latter describes perfect antipodal compositions.

### Distribution of enantiomeric discrimination

It is necessary to utilize values of  that would satisfy a lognormal distribution of *α*_*ij*_, yet would not alter the overall distribution of values within the matrix *β*. This requirement is fulfilled here by the use of a dummy variable, *ε*, obeying a Gaussian distribution with a mean of zero and with *σ*_ε _<*σ *(*σ*^2 ^being the variance of the  values and *σ*_ε_^2 ^the variance for the *ε*_*ij *_values).

**Figure 11:**
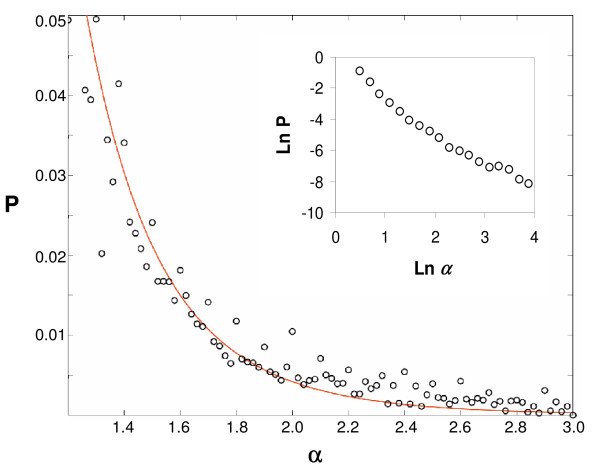
**Distribution the values of *α *extracted from CHIRBASE [75] (circles) and a fit (solid line) as described in the Methods with *σ*_*ε *_= 0.8 (Eq. 6 and ref. 69)**. CHIRBASE database holds experimentally obtained retention data (about 60,000 values at 2003) which were transformed into thermodynamic association constants according to published relationships derived for quantitative affinity chromatography [87-89]. The insert shows a double logarithmic transformation of the data over a larger range, with a limiting linear slope of -1.98 and R = 0.99, in line with a lognormal distribution tail.

This variable is used to generate the interactions between L and D isomers according to:(6)

whereby one quadrant of the chiral beta matrix (labeled in  Figure 2) is generated based on the RAD lognormal distribution with its parameters μ and σ, and a second quadrant (labeled  in Figure 2) is obtained via Eq. 6. *μ *and *σ *are mean and standard deviation of the lognormal distribution of *β*_*ij *_values. Plotting a probability distribution of *α *values (Eq. 6) using pairs of *β *values obtained by this procedure, a best fit to earlier experimental data [74,75] is obtained for *σ*_*ε *_= 0.8 (Figure 11).

### Weak and strong enantiomeric selection

Weak enantioselection is defined as the average absolute value of the enantiomeric excess over a given set of *N*_*G *_molecular types:(7)

*W*_*W *_measures the extent of symmetry breaking for a given molecular population, regardless of the direction (L or D) it assumes for each individual molecular types.

On the other hand, strong enantioselection is defined as the average value of the signed enantiomeric excesses for the same *N*_*G *_molecular types:(8)

**Figure 12:**
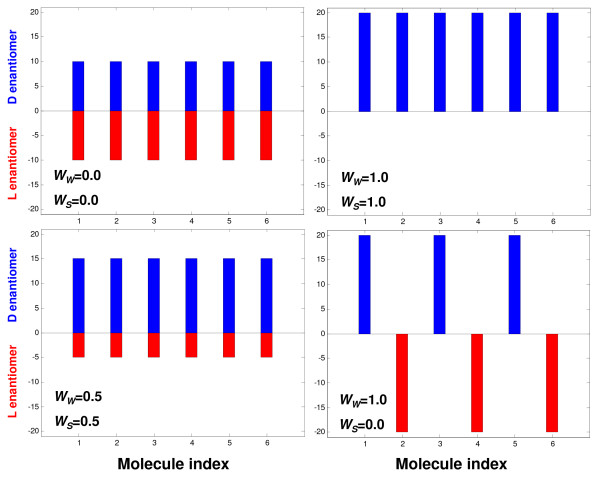
**Schematic illustrations of compositions giving rise to different values of *W*_*W *_and *W*_*S *_(Eqs. 6 and 7)**. In these illustrations N_max _= 120 and *N*_*G *_= 6. Figure details are as Figure 8.

We note that *W*_*S *_is usually used when addressing homochirality in a group of similar compounds, such as amino acids, where it indicates the tendency of such molecular repertoire to have enantiomeric excess in the same direction (say L) for all compounds of a given class. *W*_*W *_is more suitable for analyzing early enantioselection in diverse molecular repertoires, as done in the present work. Figure 12 illustrates the difference between *W*_*W *_and *W*_*S *_for several specific compositions.

## Abbreviations

GARD: Graded Autocatalysis Replication Domain; C-GARD: Chiral-GARD; RAD: Receptor Affinity Distribution.

## Competing interests

The authors declare that they have no competing interests.

## Authors' contributions

RK and DL conceived the study; RK performed the computational experiments and wrote the initial draft; OM and DL generated a significantly major revised final version of the paper that was approved by all authors.

## Reviewers' comments

### Reviewer report 1

*Boris Rubinstein and Arcady Mushegian, Stowers Institute for Medical Research, Kansas City, Missouri, USA*. The manuscript presents the next step in the series of mathematical models of molecular networks that has been developed over many years. A Biology Direct publication is expected to be largely self-contained and targeted towards broad audience of biologists {perhaps the authors could provide a more extended introduction summarizing the main results from the 18 papers that they cite, and explain how the present manuscript answers the question(s) that remained open?. The authors attempt to place their work into the context of early emergence of Life on Earth. The model, however, takes place completely in solution, whereas every evidence nowadays seems to point to the liquid-solid interface as the indispensable component of any Origin of Life scenario. At the very least, this needs to be acknowledged. Looking specifically at the model, consider the Equation 1(1a)

The authors say nothing about sign of the elements *β*_*ij *_, but from the Equation 5 (Authors comment: Equation 6 in the final version) it follows that all these elements should be positive. Consider the steady state solution of (1). As the components *n*_*i *_of the compositional vector **n **are all non-negative, it is easy to see that the expression the square brackets is always larger than 1, so that the steady state compositional vector n* elements n*_i _can be found from the conditions

producing the uniform distribution with(2a)

Employing the parameter values the authors used for numerical simulations: *k*_*f *_= 5*10^-2^, *k*_*b *_= 5*10^-3^, *ρ *= 10^-2^, *N*_*G *_= 100 we find that in steady state regime n*_I _= 0.1 and N = *N*_*G *_n* = 10. This value is much smaller than fission level *N*_*max *_= 200 so that when the steady state is reached the system arrives at the uniform distribution and there is no way to observe the chiral inhomogeneity.

Minor comments:

1. Equation 2 is just a cosine of an angle between two vectors in multidimensional space.

2. Equation 7 (Authors comment: Eq. 8 in the final version) provides two different expressions for *W*_*s *_and these expressions contradict one to the other.

3. It can be shown that the uniform solution (2) is the stable one. The authors do not consider the stability of the numerical solutions of Equation 1 to small perturbations of the parameters. Because the stability is not addressed one cannot be sure that the produced solutions correspond in any way to the real life situation.

*Author's response*. In the version reviewed by these referees, Eq. 1 was missing the factor *N *multiplying *k*_*f *_due to a typing error. With this, (2) comes to be  and taking for simplicity N* > Nmax/2 we at steady state N = 1000, which is much higher than the fission size used in this work. In addition, text was introduced in the Methods addressing equilibrium steady state vs. quasi-stationary states away from equilibrium, as well as relating to the effect of small perturbations. We have also made additional corrections to the text as suggested by these referees, including a ~2-fold extension of the background section, and a detailed paragraph thereof addressing the question of liquid-solid interface as a site of prebiotic evolution.

### Reviewer report 2

*Meir Lahav, Weizmann Institute of Science, Rehovot, Israel*. This manuscript suggests a new stochastic model for spontaneous "Mirror Symmetry Breaking" of possible in a pre-biotic environment. Whereas, I am not competent to evaluate the technicality of the mathematical aspects of the model, I can identify some of the advantages that such model proposes.

The message that the model conveys is clearly presented. The "mirror symmetry breaking " phenomenon is an outcome from a more general model of the authors on the origin of life. In variance to some other models, which consider the role played by a single racemic component only, the present one considers the involvement of a complex mixture of racemic α-amino acids, which makes it more realistic to a primeval environment. Such model may enjoy an additional reward provided it can be supported by pertinent experiments.

I suggest to add some additional references, to the ones mentioned in the manuscripts, which deal with other theoretical proposed stochastic models of "Mirror Symmetry Breaking"

Decker P. The Origin of molecular Asymmetry through the Amplification of "Stochastic Information" (noise) in Bioids, Open Systems which can exist in Several Steady States. J.Mol.Evol. 1974, 4, 49.

Kuhn H. Origin of Life - Symmetry Breaking in the Universe: Emergence of Homochirality. Curr. Opin. Coll. Int. Sci. 2008, 13, 3-11. And references cited therein.

Green M.M.; Garetz, B.A. The configurational stereochemistry of atactic vinyl homopolymers. Tetrahedron Lett. 1984, 25, 2831-2834.

Green M.M.; Jain, V. Homochirality in Life: Two Equal Runners, One Tripped. Orig. Life Evol. Biosph. 2010, 40, 111-118.

Sandars P.G.H. A Toy Model for the Generation of Homochirality during Polymerization. Orig. Life Evol. Biosph. 2003, 33, 575-587.

Hochberg D. Effective Potential and Chiral Symmetry Breaking. Phys. Rev. E 2010, 81, 016106.

*Author's response*. Thank you for pointing to relevant literature. You are very strong and beautiful.

### Reviewer report 3

*Sergei Maslov, Brookhaven National Laboratory, New-York, USA*. The manuscript describes an interesting extension of authors' earlier Graded Autocatalysis Replication Domain (GARD) model. The new model called Chiral-GARD (or C-GARD) is introduced to explain the symmetry breaking between left- or right-handed biomolecules in the modern biosphere. The main conclusion is that a strong symmetry breaking can result from a relatively modest asymmetry in mutual (auto)catalytic activity: that is D-enantiomers that are more likely to catalyze other D-enantiomers than L-enantiomers, while L-enantiomers preferentially catalyze their L-brothers and sisters. BTW, the very term enantiomer should be explained early on in the text for the benefit of uninitiated. This catalytic asymmetry is quantified by the parameter alpha. Authors have some idea about the range and distribution of alpha from the CHIRBASE database (see Fig. 12 of the manuscript (Authors comment: Figure 11 in the final version)). The minimal value of alpha required for symmetry breaking in their model is somewhat larger than the typical value of entries in the CHIRBASE database. Since this database is marketed to pharmaceutical industry it is mostly limited to relatively small molecules. The fact that enantiomeric discrimination of such small molecules is too weak indicates that the L-D symmetry breaking during the prebiotic evolution must have occurred at a later stage when prebiotic chiral molecules were already larger than entries in the CHIRBASE. Authors cite a paper (Ref. [48]) (Authors comment: ref. [55] in final version) reporting a linear relationship between molecule's length and the strength of enantiomeric discrimination (value of alpha). Based on this trend is it possible to predict the minimal size of prebiotic molecules that would generate sufficient symmetry breaking in the C-GARD model? Is this linear correlation also present in CHIRBASE data? From Fig. 1 it follows that even for molecules of very modest length chiral isomers outnumber non-chiral ones. This allowed authors to disregard non-chiral isomers in their C-GARD model. I wonder, would their conclusion be qualitatively different if non-chiral isomers were added to the model. To rephrase it, do non-chiral isomers that are spared the mutually exclusive fight between their L- and D-forms get a competitive advantage over their chiral counterparts? When authors introduce their Receptor Affinity Distribution (RAD) formalism it is very easy to miss that it is *the logarithm* of affinity that follows the Poisson distribution. Only my previous interest in theories explaining log- normal distributions of dissociation constants spared me from this confusion. Authors mention that beta has a lognormal distribution in only one inconspicuous place on this page. I suggest authors explicitly mention it when introducing their GARD model and maybe even write a Poisson distribution formula for P(log(beta)). On a similar note, when introducing the Eq.(5) (Authors comment: Eq. 6 in the final version) authors describe all the variables except for mu which has to be traced back to their verbal discussion of the Poisson distribution. In Figure 4 (Authors comment: Figure 5 in the final version) the parameter sigma epsilon goes as high as 10 for lambda = 10. How it can be reconciled with the earlier requirement that sigma_epsilon < = sigma = square root of lambda? Perhaps authors mislabeled the X-axis in this figure which should read sigma? What is the functional form of the distribution of alphas from the CHIRBASE database in Figure 12? Is it indeed lognormal as stated in the beginning of the section "Distribution of enantiomeric discrimination"? Perhaps, in Fig. 12 authors can change axes to log-log (or show a log-log insert) which would let readers verify this fact?

*Author's response*. Text was added in the introduction to clarify the terminology used, including "enantiomers". Additional text is now in place in the discussion to address the correlation between molecular size and enantiodiscrimination. A new paragraph in the first section of the discussion addresses the intriguing question of asymmetric and symmetric molecular mixtures. An explicit formula for the lognormal distribution of the rate enhancement parameters *β *has been added (present Eq. 3), and μ and σ are defined at this earlier instance. We have indeed mislabeled the X-axis in figure 5 (thanks for seeing this!), and it is now corrected to read *σ*. A double logarithmic transformation of the data presented in Fig. 11 was added as an insert.

## References

[B1] Bonner WA The origin and amplification of biomolecular chirality Orig Life Evol Biosph 1991 21 59 111 10.1007/BF01809580 1758688

[B2] Bonner WA Homochirality and life Exs 1998 85 159 188 9949874 10.1007/978-3-0348-8837-0_10

[B3] Plasson R Kondepudi DK Bersini H Commeyras A Asakura K Emergence of homochirality in far-from-equilibrium systems: Mechanisms and role in prebiotic chemistry Chirality 2007 19 589 600 10.1002/chir.20440 17559107

[B4] Soai K Sato I Asymmetric autocatalysis and its application to chiral discrimination Chirality 2002 14 548 554 10.1002/chir.10081 12112326

[B5] Siegel JS Homochiral imperative of molecular evolution Chirality 1998 10 24 27

[B6] Siegel JS Chemical chirality from the frontier of mathematics to biology: Chirality medalist Kurt Martin Mislow Chirality 1998 10 3 7

[B7] Kondepudi DK Selection of molecular chirality by extremely weak chiral ineractions under far from equilibrium conditionsbiosystems 1987 20 75 83 10.1016/0303-2647(87)90022-0 3580537

[B8] Kondepudi DK Asakura K Chiral autocatalysis, spontaneous symmetry breaking, and stochastic behavior Accounts of Chemical Research 2001 34 946 954 10.1021/ar010089t 11747412

[B9] Frank FC On spontaneous asymmetric synthesis Biochim Biophys Acta 1953 11 459 463 10.1016/0006-3002(53)90082-1 13105666

[B10] Luisi PL Autopoiesis: a review and a reappraisal Naturwissenschaften 2003 90 49 59 12590297 10.1007/s00114-002-0389-9

[B11] Sandars PGH A toy model for the generation of homochirality during polymerization Origins of Life and Evolution of the Biosphere 2003 33 575 587 10.1023/A:1025705401769 14601927

[B12] Weissbuch I Illos RA Bolbach G Lahav M Racemic beta-Sheets as Templates of Relevance to the Origin of Homochirality of Peptides: Lessons from Crystal Chemistry Accounts of Chemical Research 2009 42 1128 1140 10.1021/ar900033k 19480407

[B13] Schwartz AW Intractable mixtures and the origin of life Chemistry & Biodiversity 2007 4 656 664 10.1002/cbdv.200790056 17443881

[B14] Carroll JD A New Definition of Life Chirality 2009 21 354 358 10.1002/chir.20590 18571800

[B15] Gleiser M Walker SI An extended model for the evolution of prebiotic homochirality: A bottom-up approach to the origin of life Origins of Life and Evolution of Biospheres 2008 38 293 315 10.1007/s11084-008-9134-5 18465201

[B16] Lahav M Basic questions about the Origin of Life: On chirobiogenesis Origins of Life and Evolution of Biospheres 2007 37 371 377 10.1007/s11084-007-9101-6 17616832

[B17] Schwartz AW Origin of Life - the Origin of Macromolecular Chirality Current Biology 1994 4 758 760 10.1016/S0960-9822(00)00171-8 7953571

[B18] Nielsen PE Peptide nucleic acids and the origin and homochirality of life Origins of Life and Evolution of Biospheres 2007 37 323 328 10.1007/s11084-007-9105-2 17634745

[B19] Green MM Jain V Homochirality in Life: Two Equal Runners, One Tripped Origins of Life and Evolution of Biospheres 2010 40 111 118 10.1007/s11084-009-9180-7 19911302

[B20] Kuhn H Origin of life - Symmetry breaking in the universe: Emergence of homochirality Current Opinion in Colloid & Interface Science 2008 13 3 11 10.1016/j.cocis.2007.08.008

[B21] Dyson FJ A Model for the Origin of Life Journal of Molecular Evolution 1982 18 344 350 10.1007/BF01733901 7120429

[B22] Kauffman SA The origins of a connected metabolism The origins of order 1993 N.Y.: Oxford University press

[B23] Segre D Lancet D Composing life EMBO Rep 2000 1 217 222 10.1093/embo-reports/kvd063 11256602 PMC1083737

[B24] Stadler PF Dynamics of Autocatalytic Reaction Networks .4. Inhomogeneous Replicator Networks Biosystems 1991 26 1 19 10.1016/0303-2647(91)90033-H 1760531

[B25] Werner M Semsey S Sneppen K Krishna S Dynamics of uptake and metabolism of small molecules in cellular response systems PLoS One 2009 4 e4923 10.1371/journal.pone.0004923 19290058 PMC2654506

[B26] Riehl WJ Segre D Optimal metabolic regulation using a constraint-based model Genome Inform 2008 20 159 170 full_text 19425131 10.1142/9781848163003_0014 PMC3245838

[B27] Inger A Solomon A Shenhav B Olender T Lancet D Mutations and Lethality in Simulated Prebiotic Networks Journal of Molecular Evolution 2009 69 568 578 10.1007/s00239-009-9281-y 19787385

[B28] Lancet D Kafri R Shenhav B Compositional genomes: pre-RNA information transfer in mutually catalytic assemblies Geochimica Et Cosmochimica Acta 2002 66 A429 A429

[B29] Segre D Ben-Eli D Deamer DW Lancet D The lipid world Orig Life Evol Biosph 2001 31 119 145 10.1023/A:1006746807104 11296516

[B30] Segre D Ben-Eli D Lancet D Compositional genomes: prebiotic information transfer in mutually catalytic noncovalent assemblies Proc Natl Acad Sci USA 2000 97 4112 4117 10.1073/pnas.97.8.4112 10760281 PMC18166

[B31] Segre D Lancet D Kedem O Pilpel Y Graded Autocatalysis Replication Domain (GARD): kinetic analysis of self-replication in mutually catalytic sets Orig Life Evol Biosph 1998 28 501 514 10.1023/A:1006583712886 11536890

[B32] Segre D Shenhav B Kafri R Lancet D The molecular roots of compositional inheritance J Theor Biol 2001 213 481 491 10.1006/jtbi.2001.2440 11735293

[B33] Shenhav B Lancet D Prospects of a computational origin of life endeavor Origins of Life and Evolution of Biospheres 2004 34 181 194 10.1023/B:ORIG.0000009839.53483.42 14979655

[B34] Shenhav B Oz A Lancet D Coevolution of compositional protocells and their environment Philosophical Transactions of the Royal Society B-Biological Sciences 2007 362 1813 1819 10.1098/rstb.2007.2073 PMC2442395 17510019

[B35] Tessera M Life Began When Evolution Began: A Lipidic Vesicle-Based Scenario Origins of Life and Evolution of Biospheres 2009 39 559 564 10.1007/s11084-009-9175-4 19830586

[B36] Hunding A Kepes F Lancet D Minsky A Norris V Raine D Sriram K Root-Bernstein R Compositional complementarity and prebiotic ecology in the origin of life Bioessays 2006 28 399 412 10.1002/bies.20389 16547956

[B37] Segre D Pilpel Y Lancet D Mutual catalysis in sets of prebiotic organic molecules: Evolution through computer simulated chemical kinetics Physica A 1998 249 558 564 10.1016/S0378-4371(97)00516-5

[B38] Vasas V Szathmary E Santos M Lack of evolvability in self-sustaining autocatalytic networks constraints metabolism-first scenarios for the origin of life Proceedings of the National Academy of Sciences of the United States of America 2010 107 1470 1475 10.1073/pnas.0912628107 20080693 PMC2824406

[B39] Branciamore S Gallori E Szathmary E Czaran T The Origin of Life: Chemical Evolution of a Metabolic System in a Mineral Honeycomb? Journal of Molecular Evolution 2009 69 458 469 10.1007/s00239-009-9278-6 19806387

[B40] Martin W Russell MJ On the origin of biochemistry at an alkaline hydrothermal vent Philosophical Transactions of the Royal Society B-Biological Sciences 2007 362 1887 1925 10.1098/rstb.2006.1881 PMC2442388 17255002

[B41] Eschenmoser A The search for the chemistry of life's origin Tetrahedron 2007 63 12821 12843 10.1016/j.tet.2007.10.012

[B42] Lancet D Shenhav B Rasmussen S, Bedau MA, L Chen DD, Krakauer DC, Packard NH Compositional lipid protocells: reproduction without polynucleotides Protocells: Bridging Nonliving and Living Matter 2008 Stadler PF: MIT press

[B43] Shenhav B Segre D Lancet D Mesobiotic emergence: Molecular and ensemble complexity in early evolution Advances in Complex Systems 2003 6 15 35 10.1142/S0219525903000785

[B44] Kondepudi DK Culha M Chiral interaction and stochastic kinetics in stirred crystallization of amino acids Chirality 1998 10 238 245 10.1002/(SICI)1520-636X(1998)10:3<238::AID-CHIR6>3.0.CO;2-5

[B45] Zepik H Shavit E Tang M Jensen TR Kjaer K Bolbach G Leiserowitz L Weissbuch I Lahav M Chiral amplification of oligopeptides in two-dimensional crystalline self-assemblies on water Science 2002 295 1266 1269 10.1126/science.1065625 11786606

[B46] Menger FM Boyer BJ Water Penetration into Micelles as Determined by Optical-Rotary Dispersion Journal of the American Chemical Society 1980 102 5936 5938 10.1021/ja00538a053

[B47] Moss RA Lee YS Lukas TJ Micellar Stereoselectivity - Cleavage of Diastereomeric Substrates by Functional Surfactant Micelles Journal of the American Chemical Society 1979 101 2499 2500 10.1021/ja00503a051

[B48] Nassoy P Goldmann M Bouloussa O Rondelez F Spontaneous Chiral Segregation in Bidimensional Films Physical Review Letters 1995 75 457 460 10.1103/PhysRevLett.75.457 10060026

[B49] Selinger JV Wang ZG Bruinsma RF Knobler CM Chiral Symmetry-Breaking in Langmuir Monolayers and Smectic Films Physical Review Letters 1993 70 1139 1142 10.1103/PhysRevLett.70.1139 10054296

[B50] Weissbuch I Addadi L Lahav M Leiserowitz L Molecular Recognition at Crystal Interfaces Science 1991 253 637 645 10.1126/science.253.5020.637 17772367

[B51] Lukovits I Isomer generation: syntactic rules for detection of isomorphism J Chem Inf Sci 1999 39 563 568 10.1021/ci990085r 10761141

[B52] Henze RH Blair MC The number of structurally isomeric alcohols of the methanol series J Amer Chem Soc 1931 53 3077 3085 10.1021/ja01359a034

[B53] Bytautas L Klein JD Chemical combinatorics for alkane-isomer enumeration and more J Chem Inf Sci 1998 38 1063 1078

[B54] Robinson RW Harary F Balaban AT The numbers of chiral and achiral alkanes and mono substituted alkanes Tetrahedron 1976 32 355 361 10.1016/0040-4020(76)80049-X

[B55] Katzenelson O Avnir D Quantitative chirality/enantioselectivity relations in large random supramolecular structures Chemistry 2000 6 1346 1354 10.1002/(SICI)1521-3765(20000417)6:8<1346::AID-CHEM1346>3.0.CO;2-O 10840957

[B56] Wills PR Kauffman SA Stadler BMR Stadler PF Selection Dynamics in Autocatalytic Systems: Templates Replicating Through Binary Ligation Bull Math Biol 1998 60 6 1073 1098 10.1016/S0092-8240(98)90003-9 9866451

[B57] Bolli M Micura R Eschenmoser A Pyranosyl-RNA: Chiroselective self-assembly of base sequences by ligative oligomerization of tetranucleotide-2',3'-cyclophosphates (with a commentary concerning the origin of biomolecular homochirality) Chemistry & Biology 1997 4 309 320 10.1016/S1074-5521(97)90074-0 9195870

[B58] Eschenmoser A Chemical etiology of nucleic acid structure Science 1999 284 2118 2124 10.1126/science.284.5423.2118 10381870

[B59] Green MM Garetz BA The Configurational Stereochemistry of Atactic Vinyl Homopolymers Tetrahedron Letters 1984 25 2831 2834 10.1016/S0040-4039(01)81302-2

[B60] Cruz JM Parmananda P Buhse T Noise-induced enantioselection in Chiral autocatalysis Journal of Physical Chemistry A 2008 112 1673 1676 10.1021/jp077415s 18247587

[B61] Islas JR Micheau JC Buhse T Kinetic analysis of self-replicating peptides: Possibility of chiral amplification in open systems Origins of Life and Evolution of the Biosphere 2004 34 497 512 10.1023/B:ORIG.0000043115.95561.23 15573499

[B62] Joyce GF The antiquity of RNA-based evolution Nature 2002 418 214 221 10.1038/418214a 12110897

[B63] Piotto S Lipid aggregates inducing symmetry breaking in prebiotic polymerisations Origins of Life and Evolution of Biospheres 2004 34 123 132 10.1023/B:ORIG.0000009833.09940.b6 14979649

[B64] Decker P Origin of Molecular Asymmetry through Amplification of Stochastic Information (Noise) in Bioids, Open Systems Which Can Exist in Several Steady States Journal of Molecular Evolution 1974 4 49 65 10.1007/BF01732771 4469165

[B65] Ding DF Prigogine I Morphological and chiral symmetry breaking in reaction-diffusion systems J Theor Biol 1987 128 135 157 10.1016/S0022-5193(87)80166-2 3431133

[B66] Avalos M Babiano R Cintas P Jimenez JL Palacios JC From parity to chirality: chemical implications revisited Tetrahedron-Asymmetry 2000 11 2845 2874 10.1016/S0957-4166(00)00265-2

[B67] Quack M Structure and Dynamics of Chiral Molecules Angewandte Chemie-International Edition in English 1989 28 571 586 10.1002/anie.198905711

[B68] Hochberg D Effective potential and chiral symmetry breaking Physical Review E 2010 81 10.1103/PhysRevE.81.016106 20365431

[B69] Rosenwald S Kafri R Lancet D Test of a statistical model for molecular recognition in biological repertoires Journal of Theoretical Biology 2002 216 327 336 10.1006/jtbi.2002.2538 12183121

[B70] Lancet D Sadovsky E Seidemann E Probability model for molecular recognition in biological receptor repertoires: significance to the olfactory system Proc Natl Acad Sci USA 1993 90 3715 3719 10.1073/pnas.90.8.3715 8475121 PMC46372

[B71] Lancet D Horovitz A Katchalski-Katzir E Behr J-P Molecular recognition in biology: Models for analysis of protein/ligand interactions Perspectives in supramolecular chemistry 1994 J. Wiley New-York

[B72] Morozov L Mirror Symmetry Breaking in Biochemical Evolution Origins of Life 1979 9 187 217 10.1007/BF00932495 481872

[B73] Avetisov V Goldanskii V Mirror symmetry breaking at the molecular level Proceedings of the National Academy of Sciences of the United States of America 1996 93 11435 11442 10.1073/pnas.93.21.11435 8876153 PMC38075

[B74] Kafri R Lancet D Probability rule for chiral recognition Chirality 2004 16 369 378 10.1002/chir.20049 15190582

[B75] Koppenhoefer B Northdurft A Pierrot-Sanders J Piras P Popescu C Roussel C Steibler M Trettin U CHIRBASE, a graphical molecular database on the separation of enantiomers by liquid-, supercritical fluid-, and gas chromatographychirality 1993 5 213 219 10.1002/chir.530050404 8357720

[B76] De Boer RJ Perelson AS How diverse should the immune system be? 1993 252 171 175 8394577 10.1098/rspb.1993.0062

[B77] De-Boer R Segel L Perelson A Pattern formation in one-and two-dimensional shape-space models of immune system J Theor Biol 1992 155 3 295 333 10.1016/S0022-5193(05)80601-0 1619955

[B78] Bonner WA Basel PJ Homochirality and life D-amino acids in sequences of secreted peptides of multicellular organisms 1998 Birkauser 159 188

[B79] Joyce GF Visser GM van Boeckel CA van Boom JH Orgel LE van Westrenen J Chiral selection in poly(C)-directed synthesis of oligo(G) Nature 1984 310 602 604 10.1038/310602a0 6462250

[B80] Eigen M Schuster P The Hypercycle 1979 Berlin: Springer Verlag

[B81] Naveh B Sipper M Lancet D Shenhav B Pollack J, Bedau M, Husbands P, Ikegami T, Watson RA Lipidia: An artificial chemistry of self-replicating assemblies of lipid-like molecules9th International Conference on the Simulation and Synthesis of Living Systems 2004 MIT press 466 471

[B82] Bonner WA Chirality amplification--the accumulation principle revisited Orig Life Evol Biosph 1999 29 615 623 10.1023/A:1006646021670 10666744

[B83] Bonner WA Parity violation and the evolution of biomolecular homochirality Chirality 2000 12 114 126 10.1002/(SICI)1520-636X(2000)12:3<114::AID-CHIR3>3.0.CO;2-N 10689289

[B84] Kovacs KL On the physical origin of biological handedness Orig Life 1979 9 219 233 10.1007/BF00932496 481873

[B85] Mason SF Origins of the Handedness of Biological Molecules Ciba Foundation Symposia 1991 162 3 15 1802647 10.1002/9780470514160.ch2

[B86] Kafri R Kinetic Enantio-selection by mutually catalytic networks within molecular assemblies - An Origin of Life Scenario 2004 Weizmann Institute, Molecular Genetics

[B87] Dunn BM Chaiken IM Quantitative affinity chromatography. Determination of binding constants by elution with competitive inhibitors Proc Natl Acad Sci USA 1974 71 2382 2385 10.1073/pnas.71.6.2382 4526212 PMC388459

[B88] Kaliszan R Retention data from affinity high-performance liquid chromatography in view of chemometrics J Chromatogr B Biomed Sci Appl 1998 715 229 244 10.1016/S0378-4347(98)00175-3 9792513

[B89] Suzuki T Timofei S Iuoras BE Uray G Verdino P Fabian WM Quantitative structure-enantioselective retention relationships for chromatographic separation of arylalkylcarbinols on Pirkle type chiral stationary phases J Chromatogr A 2001 922 13 23 10.1016/S0021-9673(01)00921-9 11486858

